# Prevalence and genetic analysis of triplicated α-globin gene in Ganzhou region using high-throughput sequencing

**DOI:** 10.3389/fgene.2023.1267892

**Published:** 2023-10-19

**Authors:** Xinxing Xie, Jinhui Gan, Zezhang Liu, Yulian Zhou, Kun Yuan, Zhigang Chen, Shiping Chen, Rui Zhou, Lipei Liu, Xiaoyan Huang, Yan Zhang, Qian Liu, Wenqian Zhang, Jungao Huang, Junkun Chen

**Affiliations:** ^1^ Ganzhou Maternal and Child Health Hospital, Ganzhou, Jiangxi, China; ^2^ BGI Genomics, Shenzhen, China; ^3^ Clin Lab, BGI Genomics, Wuhan, China; ^4^ Dayu Maternal and Child Health Hospital, Ganzhou, Jiangxi, China; ^5^ Clin Lab, BGI Genomics, Tianjin, China

**Keywords:** thalassemia, α-globin gene triplication, prevalence, geographic distribution, Ganzhou, molecular diagnosis

## Abstract

α-globin gene triplication carriers were not anemic in general, while some studies found that α-globin gene triplication coinherited with heterozygous β-thalassemia may cause adverse clinical symptoms, which yet lacks sufficient evidence in large populations. In this study, we investigated the prevalence and distribution of α-globin gene triplication as well as the phenotypic characteristics of α-globin gene triplication coinherited with heterozygous β-thalassemia in Ganzhou city, southern China. During 2021-2022, a total of 73,967 random individuals who received routine health examinations before marriage were genotyped for globin gene mutations by high-throughput sequencing. Among them, 1,443 were α-globin gene triplication carriers, with a carrier rate of 1.95%. The most prevalent mutation was ααα^anti3.7^/αα (43.10%), followed by ααα^anti4.2^/αα (38.12%). 42 individuals had coinherited α-globin gene triplication and heterozygous β-thalassemia. However, they did not differ from the individuals with heterozygous β-thalassemia and normal α-globin (αα/αα) in terms of mean corpuscular volume (MCV) and mean corpuscular hemoglobin (MCH) levels. In addition, heterogenous clinical phenotypes were found in two individuals with the same genotype. Our study established a database of Ganzhou α-globin gene triplication and provided practical advice for the clinical diagnosis of α-globin gene triplication.

## 1 Introduction

Thalassemia is an autosomal recessive genetic disease with one or more mutations in globin chains. It is highly prevalent in malaria endemic areas as an adaptive response to malaria (Vlok et al., 2021). Multiple studies from Guangdong, Guangxi, Fujian, Yunnan and Guizhou provinces showed that thalassemia is highly prevalent in China with an overall carrier rate of 10.09%, and Guangxi province is the most prevalent region with a carrier rate of 19.04% (Lai et al., 2017). According to the mutated globin genes, thalassemia is mainly divided into two types: α-thalassemia and β-thalassemia (Muncie and Campbell, 2009). The imbalance between the α- and β-globin chains can result in a variety of clinical phenotypes, varying from completely healthy with normal red blood cell indices to severe anemia requiring lifelong blood transfusion. The latter poses a huge financial burden to the affected families and is one of the major public health and social problem that deserves increased efforts in investigation and prevention (Gharaibeh et al., 2018; Pepe et al., 2022).

α-globin gene triplication is the result of unequal crossing over between misaligned homologous segments in the α-globin gene cluster on chromosome 16 during meiosis, leading to an increased accumulation of α-globin (Weatherall and Clegg, 2001). In the carriers of α-globin gene triplication with a normal β-globin gene, the α-globin accumulation does not result in any clinical symptoms or significant hematological changes. However, co-inheritance of α-globin gene triplication and heterozygous β-thalassemia can increase the synthetic imbalance between α-globin and β-globin, and alter the hematological features of the carriers (Mehta et al., 2015). As a result, the co-inheritance may convert the clinical phenotype from β-thalassemia heterozygotes to β-thalassemia intermedia or β-thalassemia major (Farashi et al., 2015; Majid et al., 2015). In contrast, another study reported that there was no significant difference in the hematological indices between those co-inherited carriers and the β-thalassemia carriers with normal α-globin gene or healthy individuals (Hamid et al., 2021). Therefore, the interaction between α-globin gene triplication and heterozygous β-thalassemia is yet not clear.

Previous studies indicated that the frequencies of α-globin gene triplication in different populations varied from 0.4% (out of 125 individuals) in Sardinians, 1.2% (out of 3,500 individuals) in Netherlands to 5% (out of 50 individuals) in Greek (Goossens et al., 1980; Giordano, Bakker-Verwij, and Harteveld, 2009). In an Iran population of 4005 β-thalassemia carriers, the frequency of α-globin gene triplication was 1.67% (Hamid et al., 2021). In China, Wu et al. (2016) reported the frequency of α-globin gene triplication in 1,169 individuals from southern region was 1.2% and stressed the importance of α-globin gene triplication information in genetic counseling. More recently, the frequencies of α-globin gene triplication were found to be 0.77% (out of 7,644 individuals) in Guizhou province and 0.84% (out of 23,900 individuals) in southern Guangxi (X. Luo et al., 2021; Long and Liu, 2021). The low frequency of α-globin gene triplication in these studies suggested that a large sample size is necessary to determine the prevalence of α-globin gene triplication and its interaction with heterozygous β-thalassemia.

Jiangxi province, located in southern China, is highly prevalent with thalassemia (Zhao et al., 2018; Qiu et al., 2020). Ganzhou has been identified as the most prevalent city in southern Jiangxi with a carrier rate of 9.49%, whereas middle and northern Jiangxi has carrier rates of 3.90% and 2.63% respectively (Lin et al., 2014). Recently, a large-scale study found that the carrier rate of thalassemia in Ganzhou City is as high as 14.54% (Yang et al., 2023). However, the prevalence and distribution patterns of α-globin gene triplication, as well as its interaction with heterozygous β-thalassemia in Ganzhou city are still unknown. In this study, we performed a large-scale retrospective analysis of α-globin gene triplication in Ganzhou city based on 73,967 subjects who were genotyped using next-generation sequencing (NGS). The prevalence and distribution patterns of α-globin gene triplication were determined. In addition, we explored the interaction of α-globin gene triplication and heterozygous β-thalassemia according to the hematological parameters. This study will aid in better understanding α-globin gene triplication and providing more information for the clinical diagnosis of α-globin gene triplication.

## 2 Materials and methods

### 2.1 Population samples

A total of 73,967 subjects (37,001 males, 36,968 females, age ranged from 18 to 50 years old) receiving routine health examinations before marriage in Ganzhou city of Jiangxi province in China were genotyped for globin gene mutations including α-globin gene triplication from August 2021 to September 2022. These subjects came from 18 counties of Ganzhou, including Xingguo, Ningdu, Shicheng, Huichang, Yudu, Ganxian, Nankang, Longnan, Zhanggong, Shangyou, Chongyi, Dayu, Xinfeng, Anyuan, Xunwu, Dingnan, Quannan, and Ruijin.

### 2.2 Sample collection and blood analysis

After obtaining the informed consent of the subjects, peripheral blood was collected in EDTA tubes. All the blood samples were stored at −20°C before the experiment. The hematological parameters of all the blood samples were automatically measured using an automated XS-1000i Hematology Analyzer System (Lincolnshire, IL, United States). Mean Corpuscular Volume (MCV), Mean Corpuscular Hemoglobin (MCH) and Hemoglobin (Hb) concentration were determined and statistically analyzed using ANOVA. The relative proportions of HbA, HbA2 and HbF were measured by CAPILLARYS 3 OCTA (SEBIA, France) at Ganzhou Maternal and Child Health Hospital.

### 2.3 DNA extraction

We utilized the Q Kingfisher Flex system (Thermo Scientific, Rockford, IL) to extract genomic DNA from whole blood. Subsequently, the GenMag Nucleic Acid Isolation kit (Magnetic bead method) (GenMagBio, Beijing, China) was used for DNA isolation. To quantify the concentrations of DNA samples, we employed a NanoDrop-8000 spectrophotometer (Thermo Scientific, Waltham, MA, United States). The samples were subjected to restriction based on the following criteria: a DNA concentration exceeding 20 ng/mL and an A260/A280 ratio range of 1.8–2.0.

### 2.4 Thalassemia genotyping

We utilized a combined approach of Gap-PCR and NGS for thalassemia detection (Zhang et al., 2019). Briefly, seven types of deletion mutations (α deletions: -SEA, --THAI, -α^3.7^, -α^4.2^; β deletions: SEA-HPFH, Chinese ^G^γ^+^(^A^γδβ)^0^, Taiwanese deletion) were characterized by the Gap-PCR. Other mutations were detected using NGS. Firstly, the target sequences of *HBA1*, *HBA2,* and *HBB* were amplified and enriched through multiplex PCR. Secondly, sequencing libraries were subsequently prepared following the MGISEQ-2000 sequencing library preparation protocol (MGI, Shenzhen, China). Finally, sequencing was carried out using the paired-end tag (PE100) on an MGISEQ-2000 chip (MGI, Shenzhen, China). The protocol for bioinformatic analysis of identifying hemoglobin gene mutations was previously described (Xi et al., 2023). Furthermore, for individuals indicated as carriers of α-globin gene triplication in the analysis results, we confirmed this through PCR following the internationally recognized literature (Wang et al., 2003).

### 2.5 Multiplex ligation-dependent probe amplification

Multiplex ligation-dependent probe amplification (MLPA) was conducted with the commercial kit SALSA MLPA P140 HBA probemix (MRC-Holland, the Netherlands) by Beijing Yinda Xin Technology Co., Ltd.

### 2.6 Definition of anemia degree

According to WHO recommendations (WHO, 2011), non-anemia, mild anemia, moderate anemia and severe anemia were diagnosed using hemoglobin levels. All the subjects in this study are non-pregnant women (15 years or older) and men (15 years or older) living at altitudes lower than 1,000 m above sea level. Consequently, anemia cut-offs were 12 g/dL or higher (non-pregnant women) and 13 g/dL or higher (men) for non-anemia, 11–11.9 g/dL (non-pregnant women) and 11–12.9 g/dL (men) for mild anemia, 8–10.9 g/dL for moderate anemia, lower than 8 g/dL for severe anemia.

### 2.7 Statistical analysis

All the statistical analyses were performed using the SPSS 19 software (SPSS Inc., Chicago, IL, United States). Continuous variables were expressed as means ± SD, while categorical variables were expressed as numbers or percentages. The Chi-square test and one-way ANOVA were employed for statistical difference analysis. *p*-value <0.05 was considered as significant (*p-value <0.05; **p-value <0.01; ***p-value <0.001).

## 3 Results

### 3.1 The characteristic of α-globin gene triplication in Ganzhou

**FIGURE 1:**
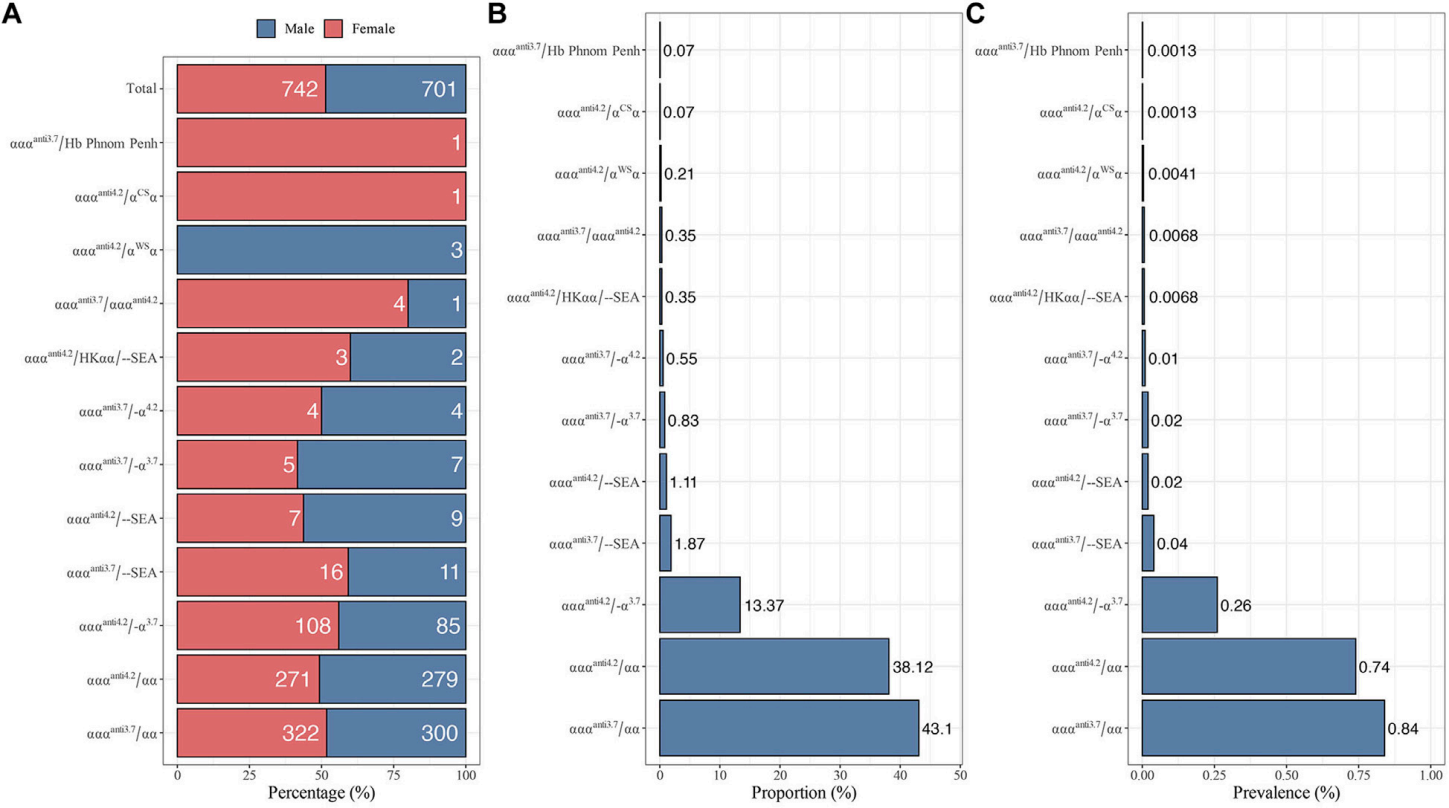
Distribution of different genotypes with α-globin gene triplication in 73,967 subjects. **(A)** Gender distribution. **(B)** The proportion of different genotypes among 1,443 α-globin gene triplication carriers. **(C)** The prevalence of different genotypes.

From 2021 to 2022, 1,443 of 73,967 subjects were diagnosed as α-globin gene triplication carriers with a carrier rate of 1.95%. There is no significant difference (*p*-value = 0.17) in the carrier rate between males (*n* = 701) and females (*n* = 742, Figure 1A). As shown in Figure 1, 12 different genotypes of α-globin gene triplications were detected. Among them, the most prevalent genotype was ααα^anti3.7^/αα, which had a carrier rate of 0.84% (622/73,967) and accounted for 43.10% (622/1,443) of the α-globin gene triplication carriers, followed by ααα^anti4.2^/αα and ααα^anti4.2^/-α^3.7^, which had carrier rates of 0.74% (550/73,967) and 0.26% (193/73,967), respectively, and accounted for 38.12% (550/1,443) and 13.37% (193/1,443) of the α-globin gene triplication carriers (Figures 1B, C). The remaining nine genotypes of ααα^anti3.7^/--SEA, ααα^anti4.2^/--SEA, ααα^anti3.7^/-α^3.7^, ααα^anti3.7^/-α^4.2^, ααα^anti4.2^/α^WS^α, ααα^anti3.7^/ααα^anti4.2^, ααα^anti4.2^/HKαα/--SEA, ααα^anti4.2^/α^CS^α, and ααα^anti3.7^/Hb Phnom Penh accounted for only 5.41% (78/1,443) of all the α-globin gene triplication carriers (Figures 1B, C). In addition, only one carrier of ααα^anti4.2^/α^CS^α and one carrier of ααα^anti3.7^/Hb Phnom were found.

### 3.2 Geographical distribution

**FIGURE 2:**
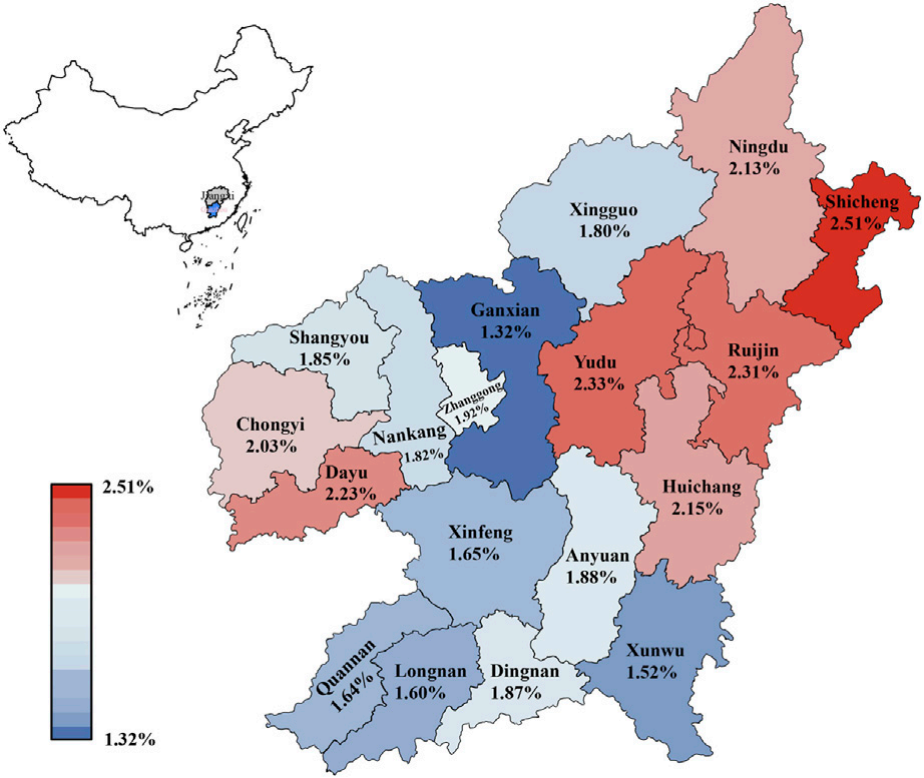
Distribution of the prevalence of α-globin gene triplication in different regions of Ganzhou city.

The carrier rates of 18 counties in Ganzhou ranged from 1.32% to 2.51% (Figure 2 and Supplementary Table S1). Shicheng county in the northeast Ganzhou had the highest carrier rate at 2.51% (63/2,514), followed by Yudu at 2.33% (241/10,351), Ruijin at 2.31% (135/5,840), Dayu at 2.23% (36/1,614) and Huichang at 2.15% (113/5256). Ganxian in the west of Ganzhou had the lowest carrier rate at 1.32% (67/5,078), which was significantly lower than Ruijin (*p* = 0.001), Yudu (*p* = 0.001), Shicheng (*p* = 0.005), Huichang (*p* = 0.012) and Dayu (*p* = 0.026), but not significantly different from Ganxian and Quannan (*p* = 0.633), Longnan (*p* = 0.480), Xunwu (*p* = 0.304), Chongyi (*p* = 0.222) and Xingguo (*p* = 0.168). Unexpectedly, in contrast to the overall distribution pattern of thalassemia observed in China, which showed high prevalence in the south and low prevalence in the north, the distribution pattern of α-globin gene triplication in Ganzhou appeared to be random.

### 3.3 Hematological parameters in the population

In order to understand the genotype-phenotype associations in α-globin gene triplication carriers, the carriers were divided into six groups (C to H) according to the genotypes of α and β globin genes (Table 1). In addition, two control groups (A and B) were randomly selected from the normal α globin (αα/αα) subjects with normal β globin (β^N^/β^N^, *n* = 80) or heterozygous β-thalassemia (β^mut^/β^N^, *n* = 80). Since all the individuals of this study were in childbearing age, the average age of each group was between 22.67 and 28.00 (Table 1). Compared with the group A with genotype αα/αα and β^N^/β^N^, the hematological parameters of group C with genotype ααα^anti3.7^/αα and β^N^/β^N^, group D with genotype ααα^anti4.2^/αα and β^N^/β^N^ and group E with genotype ααα^anti3.7^/-α^3.7^ and β^N^/β^N^ did not differ significantly, which were within the normal range (Table 1). This result suggested that α-globin gene triplication did not affect hematological parameters of the subjects with normal β globin gene at the population level. On the contrary, the MCV and MCH of group B with genotype αα/αα and β^mut^/β^N^ (MCV 66.09 ± 7.86 fL, MCH 21.15 ± 2.95 pg), group F with genotype ααα^anti3.7 or anti4.2^/αα (MCV 68.57 ± 10.22 fL, MCH 21.46 ± 3.73 pg) and β^mut^/β^N^ and group G with genotype ααα^anti4.2^/-α^3.7^ and β^mut^/β^N^ (MCV 63.47 ± 3.35 fL, MCH 20.27 ± 1.02 pg) were significantly lower than those of group A (MCV 89.51 ± 5.89 fL, MCH 29.73 ± 2.21 pg, Table 1). Moreover, the MCV and MCH of group H (ααα^anti3.7^/--SEA and β^mut^/β^N^) with only one subject were also lower than those of group A (Table 1). However, the hematological parameters of subjects in group F and G showed no significant difference in comparison with that of group B. In addition, Hb level did not vary significantly among the eight groups. These results indicated that at the population level, the α-globin gene triplication did not have large effect on the carriers in terms of hematological parameters including MCV, MCH, and Hb levels.

**TABLE 1:** Hematological parameters of samples with α gene triplication.

Group	α genotype	β genotype	n	Age	MCV (fL)	MCH (pg)	Hb (g/dL)
A	αα/αα	β^N^/β^N^	80	26.81 ± 4.63	89.51 ± 5.89	29.73 ± 2.21	13.7 ± 1.5
B	αα/αα	β^mut^/β^N^	80	27.08 ± 4.12	66.09 ± 7.86*	21.15 ± 2.95*	12.0 ± 1.5
C	ααα^anti3.7^/αα	β^N^/β^N^	429	26.50 ± 5.10	90.08 ± 8.35	29.54 ± 2.12	14.0 ± 1.6
D	ααα^anti4.2^/αα	β^N^/β^N^	368	26.90 ± 5.06	91.30 ± 9.67	29.65 ± 1.92	13.9 ± 1.9
E	ααα^anti3.7^/-α^3.7^	β^N^/β^N^	186	27.05 ± 5.56	88.54 ± 9.90	28.75 ± 3.11	13.7 ± 1.7
F	ααα^anti3.7 or anti4.2^/αα	β^mut^/β^N^	37	27.76 ± 6.83	68.57 ± 10.22*	21.46 ± 3.73*	11.4 ± 1.8
G	ααα^anti4.2^/-α^3.7^	β^mut^/β^N^	4	22.67 ± 3.06	63.47 ± 3.35*	20.27 ± 1.02*	12.5 ± 1.2
H	ααα^anti3.7^/--SEA	β^mut^/β^N^	1	28.00	66.3	20.6	11.4

β^mut^/β^N^ indicates β^0^/β^N^ or β^+^/β^N^.

### 3.4 Clinical features in subjects

To gain a further understanding of the effect of α-globin gene triplication, eight subjects with α-globin gene triplication and heterozygous β-thalassemia were successfully follow-up visited. None of them had blood transfusion history. MLPA results validated that they had triplicated α-globin genes, which was consistent with the NGS results (Supplementary Table S2 and Supplementary Figure S1). Hematological analysis revealed that all of eight subjects had Hb A ≤ 95% and Hb A2 > 3.5%, and six had MCV <80 fL and MCH <27 pg (Table 2), proving that their phenotype was β-thalassemia. According to the WHO anemia recommendations based on hemoglobin concentration, we found two subjects (5 and 8) were non-anemia, three subjects (1, 6, and 7) were mild anemia, and the remaining three subjects (2, 3, and 4) were moderate anemia (Table 2). Of note, hemoglobin concentration results showed that subjects with the same genotype had different anemic degrees, including the coinheritance of ααα^anti4.2^/αα and β^N^/β^−28 (A>G)^ (1 and 2), the coinheritance of ααα^anti3.7^/αα and β^N^/β^IVS−II−654 (C>T)^ (5 and 7) and the coinheritance of ααα^anti4.2^/αα and β^N^/β^IVS−II−654 (C>T)^ (6 and 8). These results suggested that the phenotypic impacts brought by α-globin gene triplication co-inherited with heterozygous β-thalassemia was heterogenous.

**TABLE 2:** Hematological and molecular data from carriers with α-globin gene triplication and heterozygous β-thalassemia.

Subject	Age	Sex	Genotype	MCV (fL)	MCH (pg)	Hb (g/dL)	Hb A (%)	Hb A2 (%)	Hb F (%)
1	27	F	ααα^anti4.2^/αα, β^N^/β^−28 (A>G)^	73.8	23	11.9	91.4	4.8	3.8
2	27	M	ααα^anti4.2^/αα, β^N^/β^−28 (A>G)^	89.2	31.7	10.6	94.3	5.7	0
3	21	F	ααα^anti3.7^/αα, β^N^/β^Codons41/42 (−TTCT)^	60.2	18	8.7	93.1	5.5	1.4
4	24	F	ααα^anti3.7^/αα, β^N^/β^Codons41/42 (−TTCT)^	63	19.2	10.5	93.2	5.8	1
5	26	F	ααα^anti3.7^/αα, β^N^/β^IVS−II−654 (C>T)^	85.3	28.4	13.4	93.9	4.7	1.4
6	32	M	ααα^anti4.2^/αα, β^N^/β^IVS−II−654 (C>T)^	58.4	18.4	11.2	94.9	5.1	0
7	26	M	ααα^anti3.7^/αα, β^N^/β^IVS−II−654 (C>T)^	81.8	29.8	12.9	95	5	0
8	28	M	ααα^anti4.2^/αα, β^N^/β^IVS−II−654 (C>T)^	62.8	19.8	13.6	93.3	5.1	1.6

## 4 Discussion

Since the discovery of thalassemia, pathophysiology characteristics and molecular basis of thalassemia has been studied extensively (Fibach and Rachmilewitz, 2017; Mettananda and Higgs, 2018). It is generally recognized that the imbalance between the α- and β-globin chain can result in hemoglobin disorders, such as hemoglobin H disease characterized by hemolysis with varied severities, and Bart’s Hydrops fetalis characterized by death *in utero*. However, the α-globin gene triplication was not identified until the 1980s (Higgs et al., 1980). Because α-globin gene triplication carriers are usually asymptomatic, the effects of α-globin gene triplication on thalassemia were easily ignored in epidemiological surveys (Zhuang et al., 2020; Peng et al., 2021). However, co-inheritance of α-globin gene triplication and heterozygous β-thalassemia might result in β-thalassemia intermedia (Quek and Thein, 2007). Therefore, it is necessary to investigate the prevalence of α-globin gene triplication and the phenotypic effects resulting from the co-inheritance of α-globin gene triplication and heterozygous β-thalassemia.

### 4.1 Prevalence of triplicated α-globin gene

The prevalence of α-globin gene triplication has been described in different populations. In a cohort of Sri Lanka of 620 β-thalassemia carriers, the carrier rate of α-globin gene triplication was 2.0% (Fisher et al., 2003). Another study studied 106 sickle cell patients living in the Democratic Republic of Congo, and found 11.3% patients were α-globin gene triplication carriers coinherited with α^3.7^ (Mikobi et al., 2018). In the Iranian cohort, the α-globin gene triplication carrier rate was much lower, at 0.9% in patients with sickle cell anemia and 1.67% in patients with β-thalassemia (Hamid et al., 2021). In this study, we reported the prevalence of α-globin gene triplication in Ganzhou city for the first time in a large and randomly recruited cohort of 73967 subjects. In our study, the carrier rate of α-globin gene triplication was 1.95% (1,443/73,967) (Figure 1C), which was close to the carrier rate of 2.3% of heterozygous β-thalassemia in Ganzhou city (Lin et al., 2014). Compared to previous studied, Ganzhou had a higher carrier rate of α-globin gene triplication than the Iranian population (1.2%) (Farashi et al., 2015), Dutch population (1%) (Giordano, Bakker-Verwij, and Harteveld, 2009), Guangdong province (1.2%) (Xie et al., 2015), Indians (1.1%) (Nadkarni et al., 2008) and Guizhou province (0.77%) (X. Luo et al., 2021). Unpublished data showed that the frequency of β-thalassemia carriers is 3.7% (2768/73967), of which 1.51% (42/2768) are α-globin gene triplication carriers. This frequency was slightly lower than that in Iran (1.67%) (Hamid et al., 2021). These differences were consistent with the findings that the carrier rates of the α-globin gene triplication varies among different populations (Goossens et al., 1980). Therefore, our result more accurately reflects the real prevalence of α-globin gene triplication in Ganzhou city through NGS, and 42 subjects with co-inheritance of α-globin gene triplication and heterozygous β-thalassemia were vital genetic resources for studying the influence of α-globin gene triplication on β-thalassemia.

### 4.2 Geographical distribution of triplicated α-globin gene

In China, the prevalence of thalassemia was higher in the north than in the south, which had been proved in several previous studies (Lin et al., 2014; Lai et al., 2017). In Guizhou province, the carrier rate of α-globin gene triplication was significantly higher in Qiannan (2.23%) than in other regions of Qiandongnan (1.34%), Qianbei (0.62%) and Qianxi (0.43%) (X. Luo et al., 2021), which was in accordance with the geographical distribution of thalassemia in China. Although published data showed that the carrier rate of thalassemia in the 18 counties of Ganzhou city followed this pattern (Yang et al., 2023), the distribution of α-globin gene triplication in these counties seemed to be random (Figure 1). In the south of Ganzhou, low prevalence was found in Quannan (1.64%), Xunwu (1.52%) and Xinfeng (1.65%), while higher prevalence was found in Dingnan (2.01%). In the north of Ganzhou, higher prevalence was found in Ningdu (2.01%) and Shicheng (2.39%), while lower prevalence was found in Xingguo (1.73%). These findings suggested that the distribution pattern of α-globin gene triplication was distinct from that of thalassemia and appeared to be random across the counties in Ganzhou city.

### 4.3 Interaction of triplicated α-globin gene and heterozygous β-thalassemia

Our study identified 42 carriers of both heterozygous β-thalassemia and α-globin gene triplication, providing a valuable resource to assess the interaction between heterozygous β-thalassemia and α-globin gene triplication in the Chinese population. The imbalance between α- and β-globin chains is the key to cause thalassemia. Several researchers have proposed that due to the increased α-globin accumulation, the co-inheritance of α-globin gene triplication and heterozygous β-thalassemia could result in poor clinical symptoms, such as β-thalassemia intermedia (Ma et al., 2001; Mehta et al., 2015; Ropero et al., 2022). In consistence with these studies, despite our clinical follow-up results indicated that eight carriers with both heterozygous β-thalassemia and α-globin gene triplication had no history of blood transfusion, 6 carriers exhibited anemic symptoms (subjects 1, 2, 3, 4, 6, and 7), including three moderate anemic carriers (subjects 2, 3, and 4). Moreover, the hemoglobin results revealed heterogenous anemia degrees among the subjects with identical genotype. This observation applies to subjects 1 and 2 with genotype ααα^anti4.2^/αα and β^N^/β^−28 (A>G)^, as well as subjects 6 and 8 with genotype ααα^anti3.7^/αα and β^N^/β^IVS−II−654 (C>T)^. It is intriguing to note that Camaschella et al. (1997) also observed phenotypic differences among the individuals with identical genotypes (Camaschella et al., 1997). Therefore, it is recommended to provide increased clinical attentions to the anemic individuals who had both α-globin gene triplication and heterozygous β-thalassemia.

Contrarily, a previous study conducted in an Iranian population suggested that there is no necessity to allocate additional attention to α-globin gene triplication in the carriers of heterozygous β-thalassemia (Hamid et al., 2021). Moreover, another study reported that 10 carriers of heterozygous β-thalassemia with α-globin gene triplication were asymptomatic (Mehta et al., 2015). In accordance with these findings, our findings indicated that α-globin gene triplication did not result in significant change on the mean values of hematological parameters (including MCV, MCH, and Hb levels) in either heterozygous β-thalassemia population or normal population, as demonstrated in Table 1.

We speculate that such discrepant findings observed in different studies may be attributed to the subjects analyzed. Some studies only focused on the symptomatic patients who sought medical attentions at hospitals (Ma et al., 2001; Theodoridou et al., 2020; Ropero et al., 2022), whereas our study, along with the Iranian cohort, analyzed both asymptomatic and symptomatic individuals (Hamid et al., 2021). We recommend that α-globin gene triplication should not be considered as a high-risk factor during prenatal diagnosis decisions or genetic counseling, and that the presence of α-globin gene triplication in healthy individuals should not receive extensive clinical attention. Certainly, in heterozygous β-thalassemia carriers who exhibit corresponding symptoms, clinical attention should be directed towards the potential involvement of α-globin gene triplication.

In this study, we have discovered that the α-globin gene triplication has a limited impact on the anemia phenotype. Although we thoroughly assessed all the globin gene mutations in triplication carriers, some information has not been covered, such as HBA12 which has garnered substantial attention in the research community (S.Q. Luo et al., 2020; Borgio et al., 2014). Additionally, a previous study has suggested a link between α globin gene copy number and chronic kidney disease, as well as end-stage kidney disease (Ruhl et al., 2022). Therefore, we recommend that future research endeavors continue to investigate the influence of other co-inherited variants with the triplication on the phenotype. Furthermore, it is advisable to consider monitoring the triplication’s effects on kidney function in subsequent clinical studies.

### 4.4 Conclusion

In conclusion, this study conducted an extensive survey to evaluate the occurrence and distribution of α-globin gene triplication in Ganzhou city. The results revealed that α-globin gene triplication is randomly distributed among various counties, with an overall carrier rate of 1.95%. The study also emphasized the diverse clinical manifestations of β-thalassemia with α-globin gene triplication, underscoring the importance of cautious assessment in clinical practice. These findings enrich the existing genetic data on α-globin gene triplication and offer valuable insights for diagnosing atypical cases in the future.

## Data Availability

In accordance with ethical considerations, the data presented in the current study are available from the corresponding authors upon request.
