# Supplementary material for: Prevalence and genetic analysis of triplicated α-globin gene in Ganzhou region using high-throughput sequencing
Source: Front Genet. 2023 Oct 19;14:1267892. doi: 10.3389/fgene.2023.1267892 (PMC10620506; doi:10.3389/fgene.2023.1267892)
Supplement: Supplementary file 1 [file Table1.DOCX]

Supplementary Table S1 Prevalence of α gene triplication in 18 prefectures of Ganzhou city.

| **Prefecture** | **Total number** | **Number of α triplication** | **Prevalence (%)** |
| --- | --- | --- | --- |
| **Shicheng** | 2514 | 63 | 2.51 |
| **Yudu** | 10351 | 241 | 2.33 |
| **Ruijin** | 5840 | 135 | 2.31 |
| **Dayu** | 1614 | 36 | 2.23 |
| **Huichang** | 5256 | 113 | 2.15 |
| **Ningdu** | 5031 | 107 | 2.13 |
| **Chongyi** | 1430 | 29 | 2.03 |
| **Zhanggong** | 6108 | 117 | 1.92 |
| **Anyuan** | 3240 | 61 | 1.88 |
| **Dingnan** | 1393 | 26 | 1.87 |
| **Shangyou** | 1782 | 33 | 1.85 |
| **Nankang** | 5052 | 92 | 1.82 |
| **Xingguo** | 6871 | 124 | 1.80 |
| **Xinfeng** | 5998 | 99 | 1.65 |
| **Quannan** | 1038 | 17 | 1.64 |
| **Longnan** | 2871 | 46 | 1.60 |
| **Xunwu** | 2500 | 38 | 1.52 |
| **Ganxian** | 5078 | 67 | 1.32 |
