# Supplementary figures and images for: Prevalence and genetic analysis of triplicated α-globin gene in Ganzhou region using high-throughput sequencing
Source: Front Genet. 2023 Oct 19;14:1267892. doi: 10.3389/fgene.2023.1267892 (PMC10620506; doi:10.3389/fgene.2023.1267892)

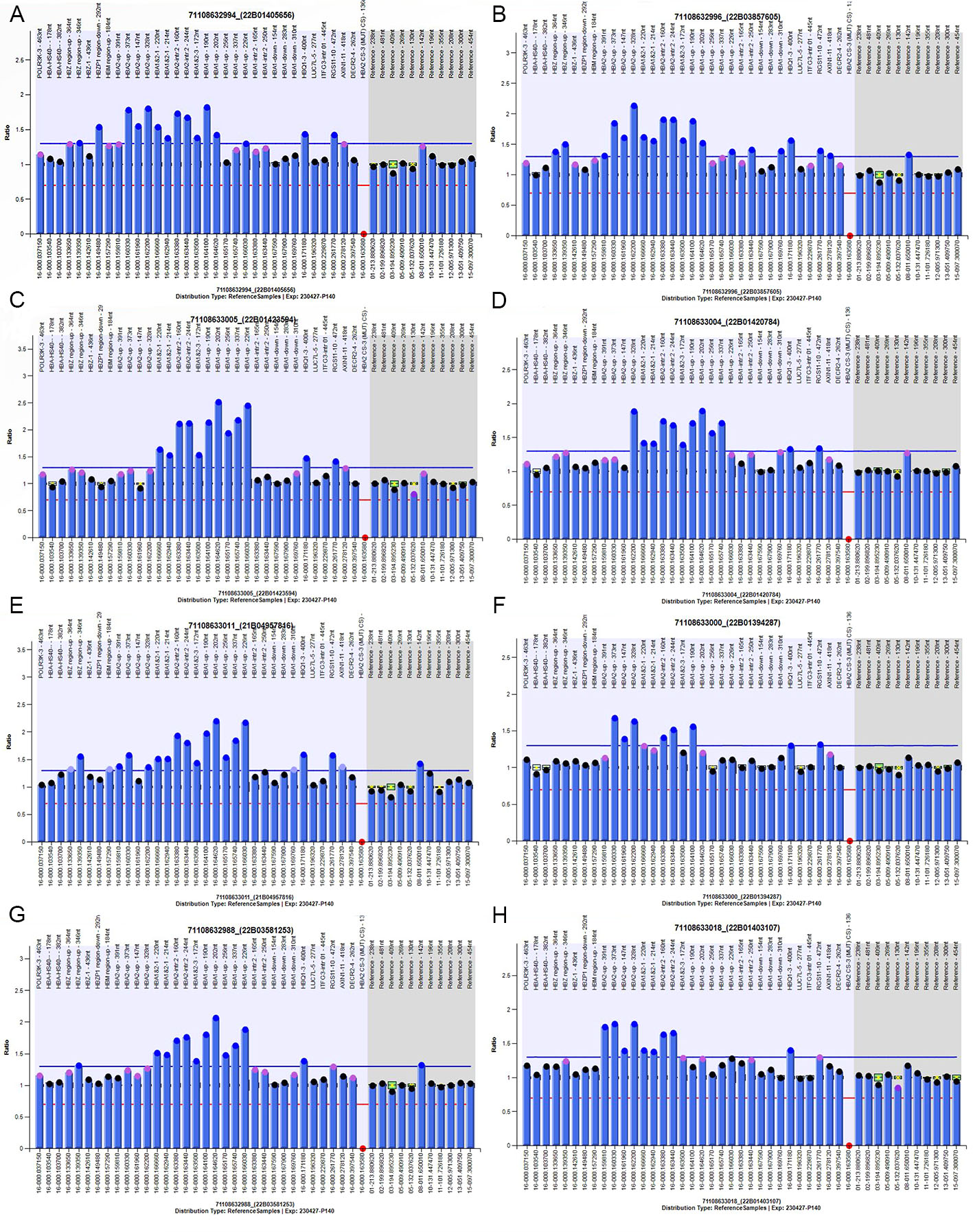

Supplement: Supplementary file 2 [file Image1.JPEG]
