# Supplementary material for: Prevalence and genetic analysis of triplicated α-globin gene in Ganzhou region using high-throughput sequencing
Source: Front Genet. 2023 Oct 19;14:1267892. doi: 10.3389/fgene.2023.1267892 (PMC10620506; doi:10.3389/fgene.2023.1267892)
Supplement: Supplementary file 3 [file Table2.DOCX]

Supplementary Table S2 Basic information of eight subjects who accepted follow-up.

| **Subject** | **Age** | **Sex** | **Triplication type (NGS)** | **Triplication type (MLPA)** |
| --- | --- | --- | --- | --- |
| **1** | 27 | F | ααα^anti4.2^ | ααα^anti4.2^ |
| **2** | 27 | M | ααα^anti4.2^ | ααα^anti4.2^ |
| **3** | 21 | F | ααα^anti3.7^ | ααα^anti3.7^ |
| **4** | 24 | F | ααα^anti3.7^ | ααα^anti3.7^ |
| **5** | 26 | F | ααα^anti3.7^ | ααα^anti3.7^ |
| **6** | 32 | M | ααα^anti4.2^ | ααα^anti4.2^ |
| **7** | 26 | M | ααα^anti3.7^ | ααα^anti3.7^ |
| **8** | 28 | M | ααα^anti4.2^ | ααα^anti4.2^ |
